# Induction of Tumor Cell Death through Targeting Tubulin and Evoking Dysregulation of Cell Cycle Regulatory Proteins by Multifunctional Cinnamaldehydes

**DOI:** 10.1371/journal.pone.0050125

**Published:** 2012-11-21

**Authors:** Amrita A. Nagle, Fei-Fei Gan, Gavin Jones, Choon-Leng So, Geoffrey Wells, Eng-Hui Chew

**Affiliations:** 1 Department of Pharmacy, National University of Singapore, Singapore, Republic of Singapore; 2 Department of Pharmaceutical and Biological Chemistry, University College London School of Pharmacy, London, United Kingdom; Enzo Life Sciences, Inc., United States of America

## Abstract

Multifunctional *trans*-cinnamaldehyde (CA) and its analogs display anti-cancer properties, with 2-benzoyloxycinnamaldehyde (BCA) and 5-fluoro-2-hydroxycinnamaldehyde (FHCA) being identified as the *ortho*-substituted analogs that possess potent anti-tumor activities. In this study, BCA, FHCA and a novel analog 5-fluoro-2-benzoyloxycinnamaldehyde (FBCA), were demonstrated to decrease growth and colony formation of human colon-derived HCT 116 and mammary-derived MCF-7 carcinoma cells under non-adhesive conditions. The 2-benzoyloxy and 5-fluoro substituents rendered FBCA more potent than BCA and equipotent to FHCA. The cellular events by which these cinnamaldehydes caused G_2_/M phase arrest and halted proliferation of HCT 116 cells were thereby investigated. Lack of significant accumulation of mitosis marker phospho-histone H3 in cinnamaldehyde-treated cells indicated that the analogs arrested cells in G_2_ phase. G_2_ arrest was brought about partly by cinnamaldehyde-mediated depletion of cell cycle proteins involved in regulating G_2_ to M transition and spindle assembly, namely cdk1, cdc25C, mad2, cdc20 and survivin. Cyclin B1 levels were found to be increased, which in the absence of active cdk1, would fail to drive cells into M phase. Concentrations of cinnamaldehydes that brought about dysregulation of levels of cell cycle proteins also caused tubulin aggregation, as evident from immunodetection of dose-dependent tubulin accumulation in the insoluble cell lysate fractions. In a cell-free system, reduced biotin-conjugated iodoacetamide (BIAM) labeling of tubulin protein pretreated with cinnamaldehydes was indicative of drug interaction with the sulfhydryl groups in tubulin. In conclusion, cinnamaldehydes treatment at proapoptotic concentrations caused tubulin aggregation and dysegulation of cell cycle regulatory proteins cdk1 and cdc25C that contributed at least in part to arresting cells at G_2_ phase, resulting in apoptotic cell death characterized by emergence of cleaved forms of caspase 3 and poly (ADP-ribose) polymerase (PARP). Results presented in this study have thus provided further insights into the intricate network of cellular events by which cinnamaldehydes induce tumor cell death.

## Introduction

The cell cycle is tightly regulated by checkpoints which ensure sequential progression through all the phases. If certain critical events in a phase cannot be executed, these checkpoints activate a “wait” signal causing cell cycle arrest until the events are completed as programmed. However, if the normal cell cycle cannot be restored, apoptotic pathways are activated [1]. Cancerous cells usually exhibit high growth rates because of deregulation of cell cycle and apoptotic signaling pathways [2]. For this reason, induction of cell cycle arrest is considered as a rational strategy to propel tumor cells into apoptosis [3].

Tubulin monomers polymerize to form microtubules that comprise an essential part of the cytoskeleton. At the onset of mitosis, the interphase microtubular network metamorphoses into the mitotic spindle that faithfully segregates the two pairs of chromosomes into daughter cells [4]. Microtubule dynamics are crucial for spindle formation, and its inhibition may trigger induction of apoptosis [5]. Certain clinically used anti-cancer agents cause suppression of microtubule dynamics either by reducing polymerization (e.g. vinblastine) or by excessive polymerization (e.g. paclitaxel), thus driving the cell into apoptosis [6], [7].

**Figure 1:**
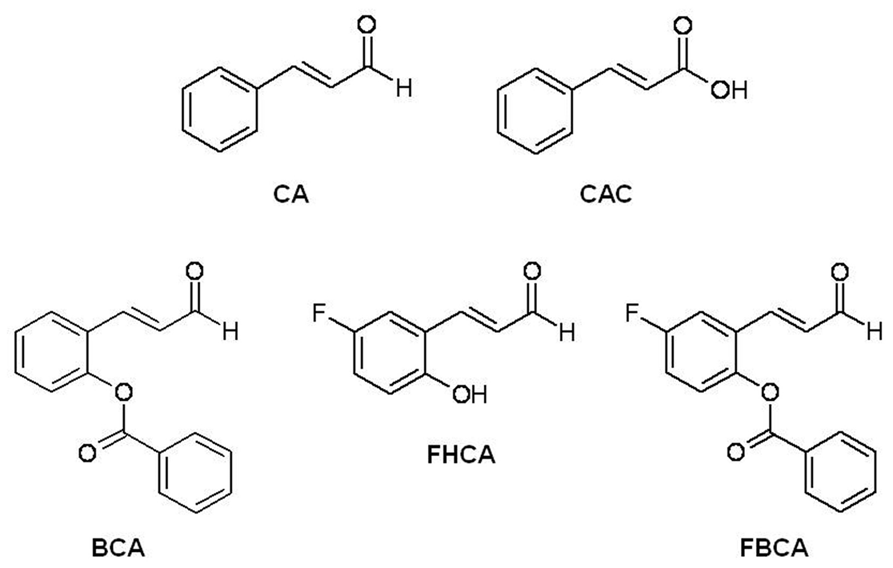
Chemical structures of cinnamaldehyde analogs and cinnamic acid (CAC).

*Trans*-cinnamaldehyde (CA), the active component in cinnamon, and its structural analogs have been reported to exhibit an array of biological activities including antiproliferative actions against cancer cells [8]–[14]. Independent studies have proposed diverse mechanisms responsible for the apoptosis inducing properties of CA and its analogs (henceforth collectively referred to as cinnamaldehydes) including loss of mitochondrial membrane potential [15], inhibition of proteasomal activity [16], inhibition of AP-1 activity [17], and production of reactive oxygen species [18], [19]. In addition, our laboratory has recently reported inhibition of thioredoxin reductase as one of the mechanisms responsible for the antiproliferative activities of cinnamaldehydes [20]. The multi-targeting nature of cinnamaldehydes and their yet-to-be fully defined proapoptotic activities have prompted the constant quest for more potent derivatives and better understanding of their molecular mechanisms. In this present study, a novel analog 5-fluoro-2-benzoyloxycinnamaldehyde (FBCA), together with 2-benzoyloxycinnamaldehyde (BCA) and 5-fluoro-2-hydroxycinnamaldehyde (FHCA), the two lead analogs identified from a previous study [20], were investigated for the mechanisms underlying their antiproliferative effects on the susceptible human-derived colon carcinoma HCT 116 cell line. Cinnamaldehydes at proapoptotic concentrations induced a G_2_/M phase arrest that was associated with tubulin aggregation. In addition, levels of key proteins regulating the G_2_/M phase including cdk1, cdc25C, cdc20, mad2 and survivin were found to be decreased, while cyclin B1 levels were increased. Taken together, the results indicated that cinnamaldehydes induced tumor cell death, at least in part, through targeting tubulin and evoking dysregulation of cell cycle regulatory proteins. This has led to a better understanding of the complex network of cellular events by which cinnamaldehydes halt tumor cell proliferation, which can further aid the development of this class of compounds as potential cancer therapeutics.

**Table 1:** Antiproliferative activities of cinnamaldehyde analogs against HCT 116, MCF-7 and MRC-5 cells.

Compounds	HCT 116		MCF-7		MRC-5	
	Mean GI_50_ a(µM)	Mean LC_50_ b(µM)	Mean GI_50_ a(µM)	Mean LC_50_ b(µM)	Mean GI_50_ a(µM)	Mean LC_50_ b(µM)
**CA** c	9.2±1.4	35.0±11.2	8.2±1.3	62.3±22.8	40.4±5.9	86.4±1.7
**BCA** c	3.5±1.5	12.1±4.1	4.2±0.9	21.7±3.1	14.3±3.8	25.7±3.8
**FHCA** c	1.6±0.3	3.2±0.5	1.9±0.2	20.7±0.4	15.2±1.4	27.5±1.9
**FBCA**	1.2±0.3	2.9±0.1	1.9±0.8	9.1±0.6	12.7±1.2	24.2±0.8

Values are presented as means±SD from 3 independent experiments.

a GI_50_∶50% growth inhibition concentration;

b LC_50_∶50% lethal concentration;

c Results for HCT 116 and MCF-7 cell lines were adopted from Ref 20.

**Figure 2:**
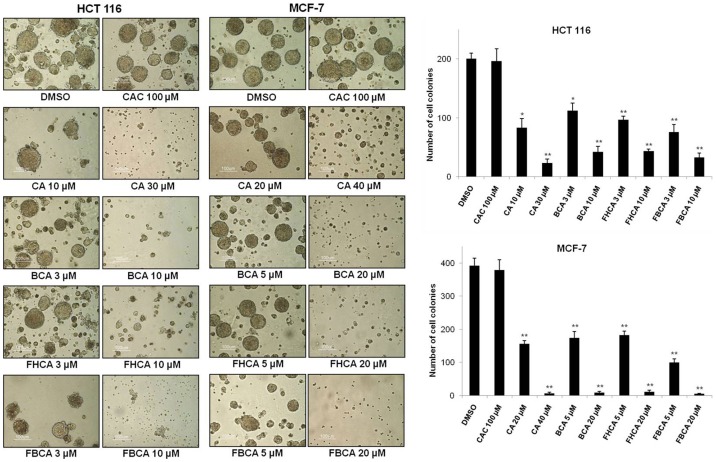
Effect of cinnamaldehydes on anchorage-independent growth of HCT 116 and MCF-7 cells. Cell suspensions prepared in RPMI 1640 agarose medium containing DMSO, CAC or different concentrations of cinnamaldehydes were layered onto a pre-solidified agarose medium in each well of 6-well plates. The cultures were incubated at 37°C for two weeks and cell colonies greater than 0.1 mm were counted manually using a light microscope. Left panel: representative photomicrographs (10× magnification) illuminating the effects of cinnamaldehydes on the anchorage-independent growth of HCT 116 and MCF-7 cells. Right panel: Bar graphs showing reduction in number of cell colonies. *p<0.05, **p<0.01 versus DMSO control.

## Materials and Methods

### Chemicals and Materials

CA, cinnamic acid (CAC) and paclitaxel were purchased from Sigma-Aldrich (St. Louis, MO). Pan caspase inhibitor Z-VAD-FMK was from R&D Systems (Minneapolis, MN). The analogs BCA and FHCA were synthesized as described previously [20]. Purified porcine brain tubulin was purchased from Cytoskeleton (Denver, CO) and biotin-conjugated iodoacetamide (N-(Biotinoyl)-N'-(Iodoacetyl)Ethylenediamine, BIAM) was purchased from Molecular Probes (Invitrogen, Eugene, OR).

**Figure 3:**
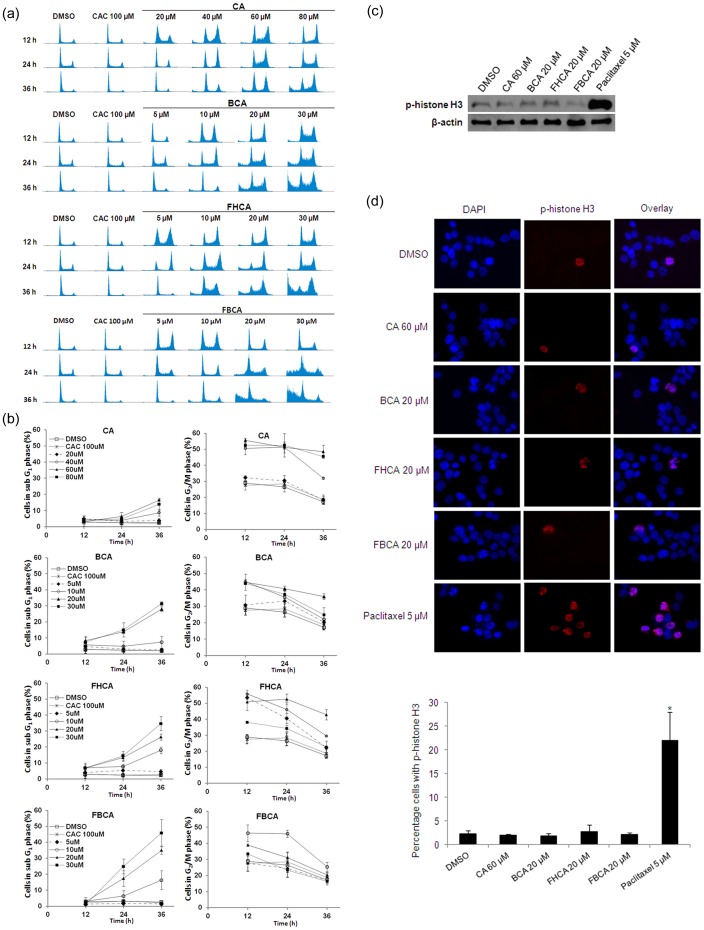
Effect of cinnamaldehydes on cell cycle progression of HCT 116 cells. (a) Representative DNA histograms of cells following treatment with indicated concentrations of CA, BCA, FHCA or FBCA at timepoints as indicated. (b) Percentages of cells in sub G_1_ and G_2_/M phases after exposure to CA, BCA, FHCA or FBCA. Data points are means±SD of three independent experiments. (c) Cells were treated with cinnamaldehydes at indicated concentrations for 12 h. Cell lysates were analyzed by Western blotting for phospho-histone H3 (p-histone H3). (d) Upper panel: Representative immunofluorescence microscopic images of cells cultured on coverslips and treated or untreated with cinnamaldehyde analogs or paclitaxel at indicated concentrations for 12 h. Following drug treatment, cells were fixed and probed with anti-phospho-histone H3 antibody followed by Alexa Fluor 594-conjugated secondary antibodies, then counterstained with DAPI. Images were obtained at 40×magnification. Lower panel: Bar graphs depicting percentage of number of cells co-stained with DAPI and Alexa Fluor 594 in a total of 6 microscopic images taken from random spots on each immunolabelled coverslips. Data points are means±SD of three independent experiments. *p<0.05 versus DMSO control.

**Figure 4:**
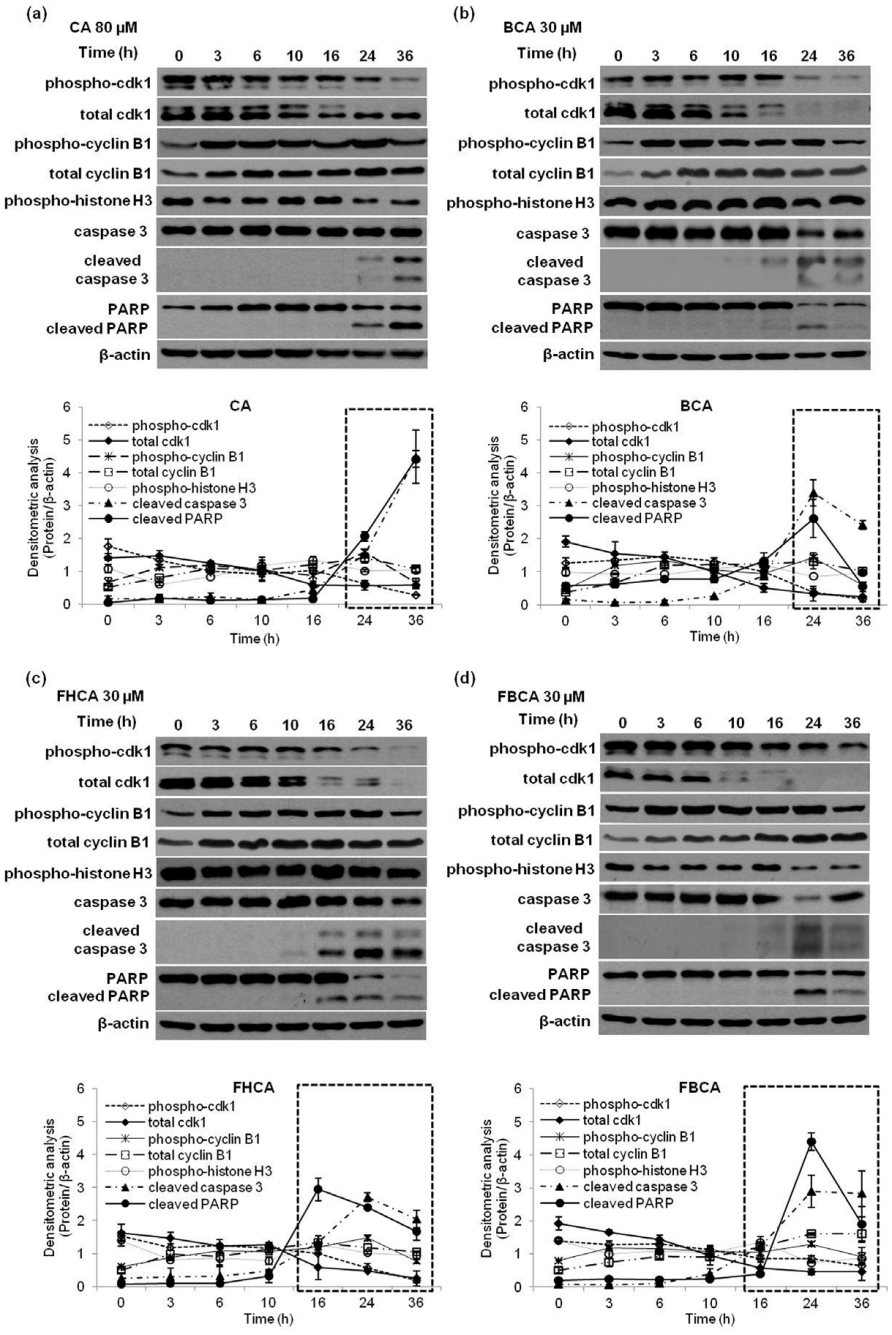
Time-dependent effect of cinnamaldehydes on cdk1, cyclin B1, phospho-histone H3, and induction of apoptosis. HCT 116 cells treated with a proapoptotic dose of cinnamaldehydes were harvested at the indicated timepoints. Cell lysates were analyzed for cdk1, phospho-cdk1, cyclin B1, phospho-cyclin B1, phospho-histone H3, caspase 3 (full length and cleaved) and PARP (full length and cleaved) using Western blot analysis. Blots were probed for β-actin to ensure equal protein loading. Bottom panels of western blot analyses for each compound: densitometric intensities of each protein normalized to that of each respective actin loading control. Results are presented as means±SD of at least two independent experiments; SD denoted by error bars. Data points at timepoints within boxed region show *p*<0.05 versus timepoint = 0.

**Figure 5:**
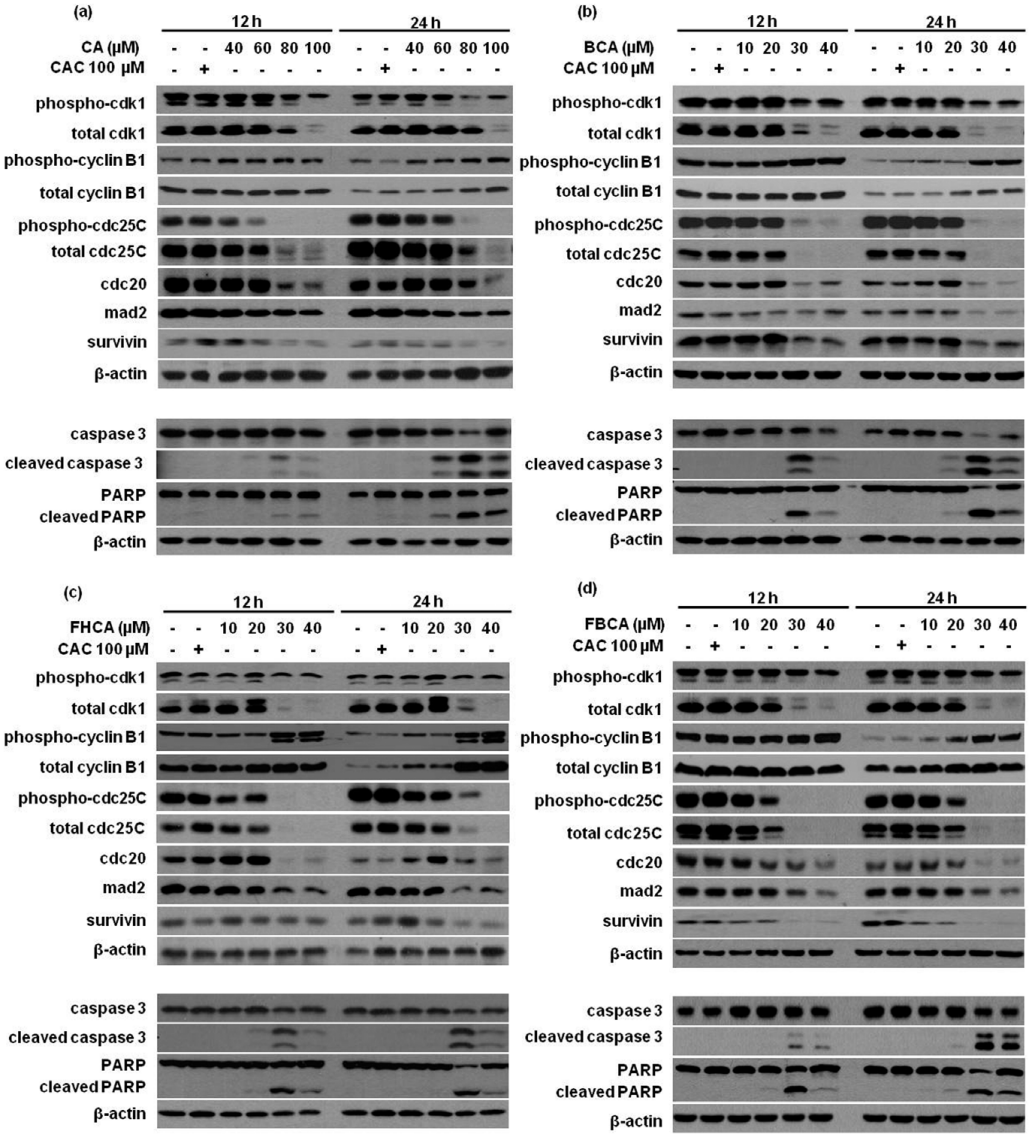
Dose-dependent effect of (a) CA, (b) BCA, (c) FHCA and (d) FBCA on cell cycle regulatory proteins. HCT 116 cells were treated with indicated concentrations of cinnamaldehydes for 12 h or 24 h. Cell lysates were analyzed by Western blotting for proteins controlling G_2_ to mitosis transition (cdk1, phospho-cdk1, cdc25C, phospho-cdc25C, cyclin B1, phospho-cyclin B1), proteins regulating spindle assembly (mad2, cdc20, survivin) and apoptotic markers (full length and cleaved) caspase 3, and (full length and cleaved) PARP. Blots were also probed for β-actin to ensure equal protein loading.

### Chemical Synthesis

#### 5-Fluoro-2-benzoyloxycinnamaldehyde [(E)-4-fluoro-2-(3-oxoprop-1-en-1-yl)phenyl benzoate]

Triethylamine (0.137 g, 1.355 mmol) was added to a solution of (*E*)-3-(5-fluoro-2-hydroxyphenyl) acrylaldehyde [21] (0.150 g, 0.903 mmol) and benzoyl chloride (0.152 g, 1.084 mmol) in dichloromethane (7 ml). The reaction mixture was stirred at room temperature for 30 min, followed by reaction quenching with water (20 ml) and extraction with dichloromethane (2×10 ml). The combined organic extracts were dried over magnesium sulphate and solvent was removed in vacuo. The residue was purified by column chromatography with hexane/ethyl acetate (9/1) to give the product as a white solid (0.234 g, 0.866 mmol, 96%). ^1^H NMR (400MHz) δ: 6.71 (d, *J* = 8.0 Hz, 1H), 7.25 (m, 2H), 7.41 (d, *J* = 7.4 Hz, 1H), 7.58 (m, 3H), 7.73 (d, *J* = 8.0 Hz, 1H), 8.25 (d, *J* = 8.0 Hz, 2H), 9.66 (s, 1H).

**Figure 6:**
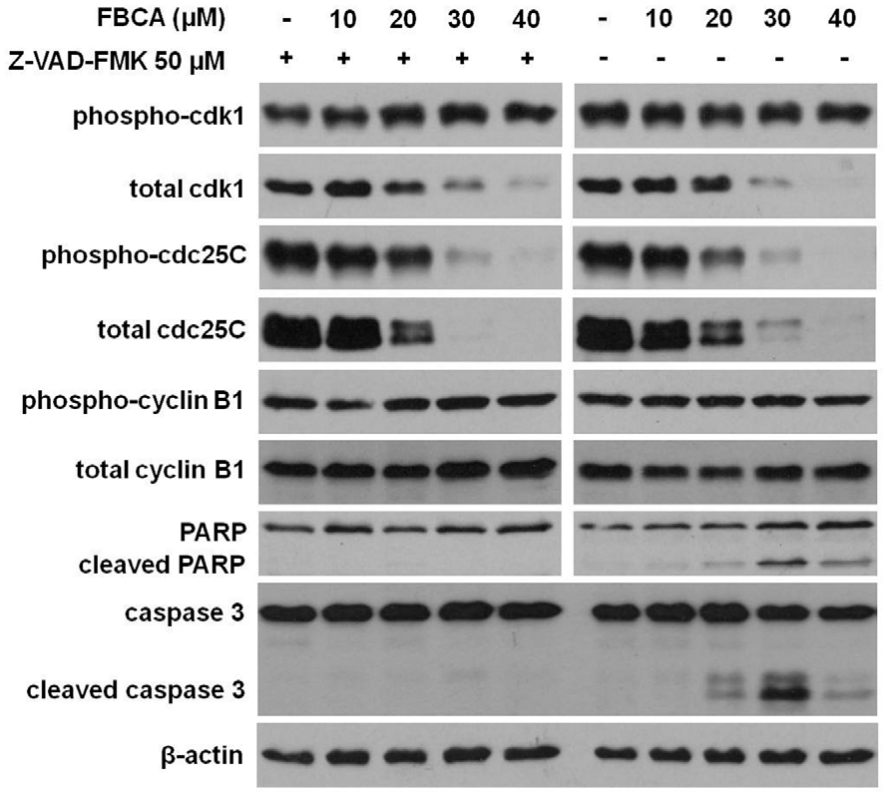
Effect of FBCA on cell cycle regulatory proteins in cells pretreated with pan caspase inhibitor Z-VAD-FMK. HCT 116 cells were treated or untreated with 50 µM Z-VAD-FMK for 1 h, followed by addition of FBCA at indicated concentrations for another 12 h. Cell lysates were analyzed by Western blotting for proteins controlling G_2_ to mitosis transition (cdk1, phospho-cdk1, cdc25C, phospho-cdc25C, cyclin B1, phospho-cyclin B1) and apoptotic markers (full length and cleaved) caspase 3, and (full length and cleaved) PARP. Blots were also probed for β-actin to ensure equal protein loading.

**Figure 7:**
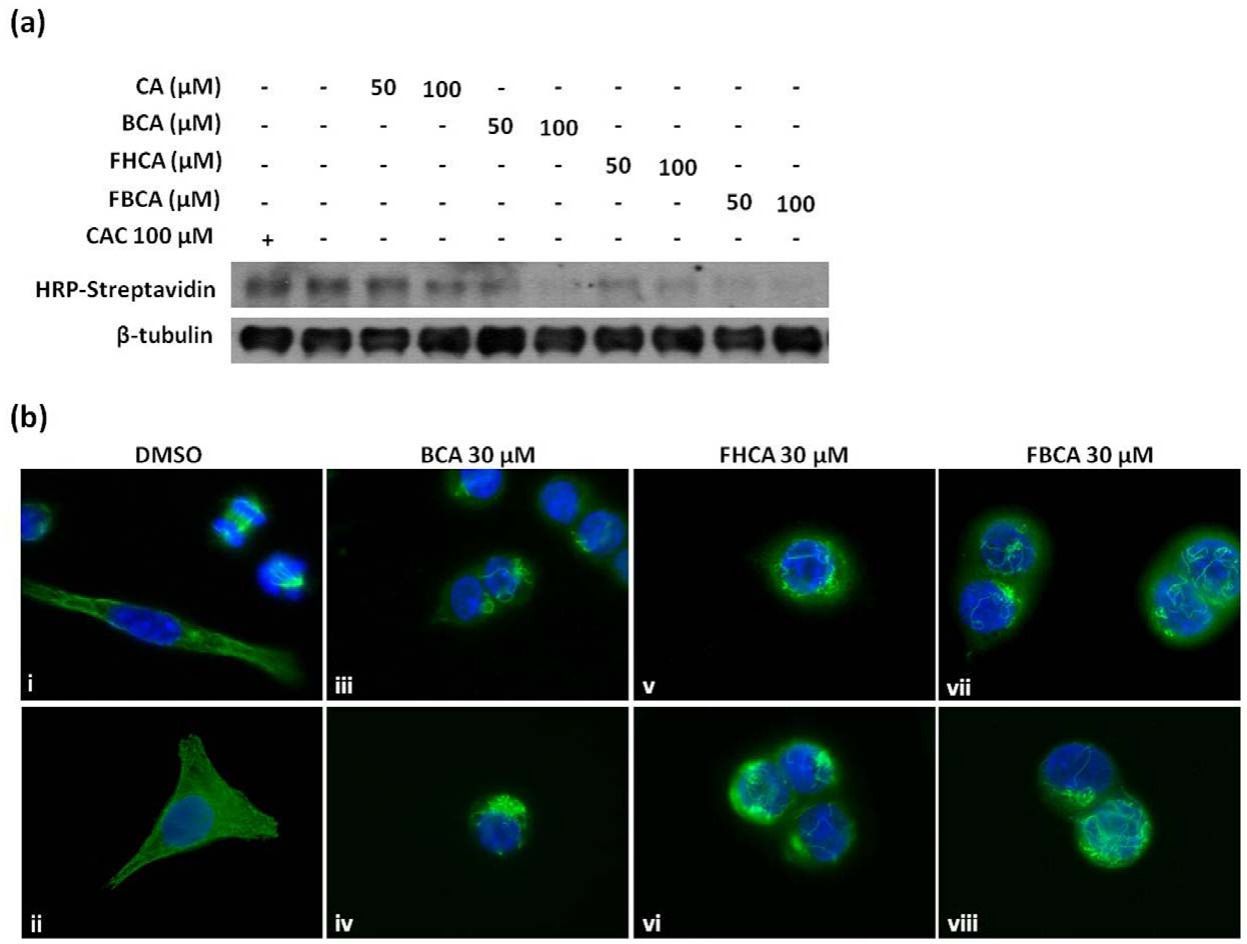
Interaction of cinnamaldehydes with sulfhydryl groups in tubulin and their effects on tubulin organization in HCT 116 cells. (a) Cinnamaldehydes were incubated with purified tubulin for 30 min at 37°C and aliquots of samples were labeled with BIAM. Western blotting by HRP-streptavidin was performed to detect BIAM labeling of free thiol groups. Equal amount of tubulin across all samples was ascertained by probing for β-tubulin. (b) Representative immunofluorescence microscopic images of cells treated with DMSO (i & ii), 30 µM BCA (iii & iv), 30 µM FHCA (v & vi), and 30 µM FBCA (vii & viii). Cells cultured on coverslips and treated with DMSO or cinnamaldehydes for 8 h were fixed, and subjected to immunocytochemical analysis using anti-β-tubulin antibody. Coverslips were then mounted on slides using mounting medium containing DAPI and images were obtained at 100×magnification.

**Figure 8:**
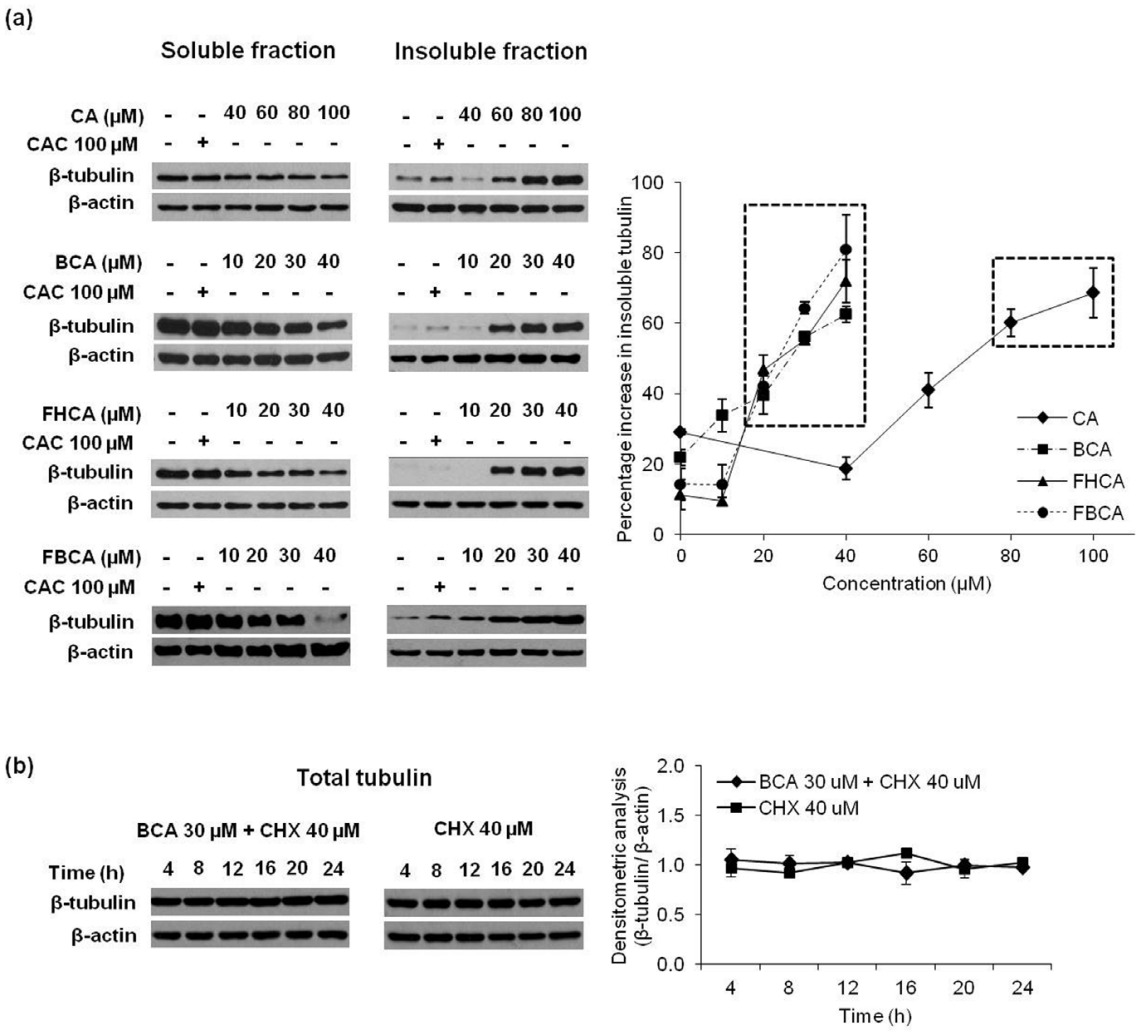
Cinnamaldehydes cause dose-dependent accumulation of insoluble tubulin. (a) HCT 116 cells were treated with cinnamaldehydes at indicated concentrations for 16 h. Soluble (left panel) and insoluble (middle panel) fractions of collected cell lysates were analyzed by Western blotting with anti-β-tubulin antibody. (b) HCT 116 cells were treated with 40 µM cycloheximide (CHX) alone or 30 µM BCA+40 µM CHX and lysates containing both soluble and insoluble tubulin were collected at indicated timepoints. Total tubulin in the lysates was analyzed by western blotting with anti-β-tubulin antibody. Blots were probed for β-actin to ensure equal protein loading. Right panel of (a): band intensities quantified by densitometry and normalized to respective actin loading were expressed as percentage of insoluble tubulin per total tubulin. Right panel of (b): band intensities were quantified by densitometry and normalized to respective actin loading control. Results are presented as means±SD of at least two independent experiments; SD denoted by error bars. Statistically significant (*p*<0.05 versus DMSO control) data points were boxed.

**Figure 9:**
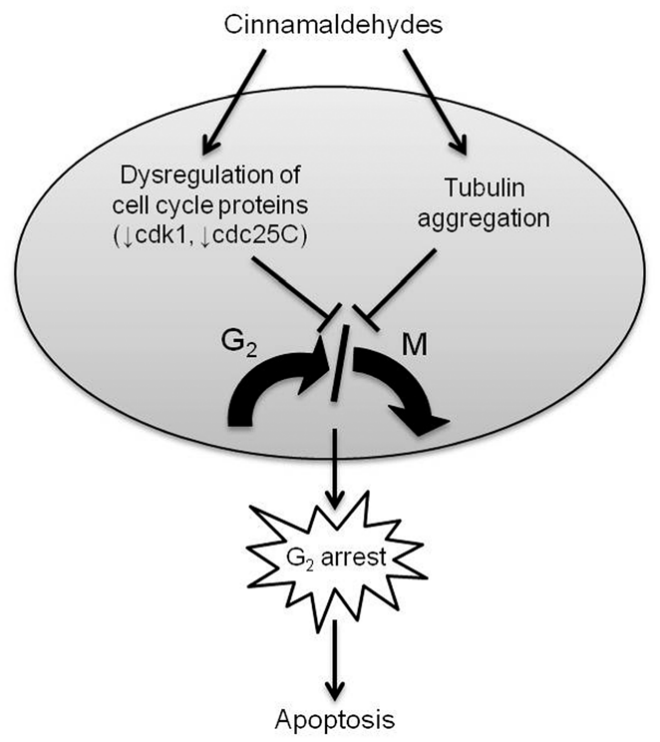
Schematic summary of the proposed cellular outcomes resulting from treatment with lethal concentrations of cinnamaldehydes. A lethal dose of cinnamaldehydes resulted in G_2_ arrest associated with down-regulation of cell cycle regulatory proteins cdk1 and cdc25C and tubulin aggregation, which prevented arrested cells from entering into M phase. This finally led to apoptosis.

### Cell Culture and Antibodies

Human-derived colon carcinoma HCT 116, breast carcinoma MCF-7 and normal lung fibroblast MRC-5 cells were obtained from ATCC (Rockville, MD). The two cancer cell lines and fibroblasts were respectively maintained in RPMI 1640 and MEM medium supplemented with 10% fetal bovine serum (FBS), 100 units/ml penicillin and 1 µg/ml streptomycin at 37°C and 5% CO_2_. Antibodies against cdc25C, mad2 and β-actin were purchased from Santa Cruz Biotechnology (Santa Cruz, CA), and those against caspase 3, cleaved caspase 3 (Asp 175), phospho-cdk1 (Tyr15), cdk1, PARP/cleaved PARP, phospho-cdc25C (Ser216), phospho-cyclin B1 (Ser133), cyclin-B1, phospho-histone H3 (Ser10) and cdc20 were obtained from Cell Signaling Technology (Beverly, MA), anti-survivin antibody was purchased from Epitomics (Burlingame, CA), and monoclonal anti-β-tubulin (T5201) antibody was from Sigma-Aldrich. Horseradish peroxidase (HRP) conjugated anti-mouse and anti-rabbit secondary antibodies were acquired from Pierce (Rockford, IL), while Alexa Fluor 488 conjugated anti-mouse IgG and Alexa Fluor 594-conjugated anti-rabbit IgG were purchased from Molecular Probes.

### Cell Viability Assay

The effect of cinnamaldehydes on cell viability was assessed by viable cell-mediated reduction of 3-(4,5-dimethylthiazol-2-yl)-2,5-diphenyltetrazolium bromide (MTT). HCT116, MCF-7 and MRC-5 cells were seeded in 96-well plates at a density of 3×10^3^, 4×10^3^ and 6×10^3^ per well respectively, allowed to attach for around 24–36 h to reach approximately 60% confluence, and treated with drugs. Drug solutions in medium were freshly prepared from drug stocks in DMSO before treatment. After 72 h of drug treatment, MTT (400 µg/ml) was added into each well and plates were incubated for another 4 h. Medium and MTT from the wells was aspirated, the purple formazan crystals were dissolved in a DMSO:Glycine buffer pH 10.5 (4∶1) mixture and absorbance was read at 550 nm.

### Soft Agar Assay

HCT 116 (3×10^4^) or MCF-7 (5×10^4^) cells were suspended in RPMI 1640 medium containing 0.33% w/v low melting agarose, 10% FBS, as well as DMSO or different concentrations of cinnamaldehydes. The cell suspensions were then layered onto a bottom layer of RPMI 1640 medium containing 0.5% agarose and 10% FBS pre-solidified in each well of six-well plates. The cells were incubated at 37°C in a humidified atmosphere containing 5% CO_2_ for 2 weeks, after which cell colonies of size bigger than 0.1 mm were enumerated at 10x magnification using a Nikon Eclipse TE2000-U microscope (Tokyo, Japan). Representative photomicrographs were taken using a Nikon Digital Camera DXM1200 connected to the microscope.

### Cell Cycle Analysis

HCT 116 cells were seeded in six-well plates at a density of 5×10^5^ cells per well and incubated for 24 h before drug treatment. Following drug treatment, floating and attached cells were collected at pre-determined timepoints, washed with cold PBS and incubated overnight in 500 µl hypotonic fluorochrome solution [50 µg/ml propidium iodide (PI), 0.1% sodium citrate, 0.1% Triton X-100, and 0.1 mg/ml RNase A] at 4°C protected from light. Samples were then analyzed on a Beckman Coulter (Dako) CyAn ADP flow cytometer and data analysis was performed using Summit Software v4.3.

### Immunocytochemistry

HCT 116 cells (5×10^5^) were cultured on coverslips (pre-coated with 0.01% w/v poly-L-lysine) placed in individual wells of six-well plates. After overnight incubation, cells were treated with drugs for 12 h, and fixed with 4% paraformaldehyde. Fixed cells were permeabilized using 2% Triton X-100 in PBS, followed by blocking with 5% horse serum, then probed with anti-phospho-histone H3 antibody followed by Alexa Fluor 594-conjugated anti-rabbit IgG or anti-β-tubulin antibody followed by Alexa Fluor 488-conjugated anti-mouse IgG. The immunolabeled coverslips were mounted on glass slides using Vectashield mounting medium with 4′,6-diamidino-2-phenylindole (DAPI; Vectorlabs, Burlingame, CA). Microscopic images were obtained using a Nikon Eclipse T*i*-S epifluorescence microscope connected to a Nikon DS-Ri1 camera. To quantify cells stained by anti-phospho-histone H3 antibody, results were expressed as a percentage of cells co-labelled with DAPI and Alexa Fluor 594 over cells stained with DAPI in a total of 6 microscopic images (40×magnification) taken from random spots on the immunolabelled coverslips. Results were representative of three independent experiments.

### Cell Lysate Preparation for Western Blot Analysis of Cell Cycle Regulatory Proteins

HCT 116 cells (1.5×10^6^) were cultured on 100 mm plates and incubated for 24 h to allow attachment and exponential growth before treatment with cinnamaldehydes. Adherent and floating cells were pelleted, washed with cold PBS and lysed in Triton X-100 lysis buffer. Protein contents were determined by modified Bradford assay.

### Western Blot Analysis

Cell lysates or their fractions containing equal amounts of protein (30 µg) were resolved by SDS-PAGE (10–15% gels) and transferred to nitrocellulose membranes (Bio-Rad laboratories, Hercules, CA). Membranes were probed with primary antibody followed by secondary antibody conjugated to HRP, and then developed using enhanced chemiluminescence system [SuperSignal West Femto, (Pierce) or Western Lightning Plus, PerkinElmer (Boston, MA)]. All blots are representative of at least two independent experiments.

### Cell Lysate Preparation for Separation of Soluble and Insoluble Tubulin Fractions

HCT 116 cells were seeded on 60 mm plates at a density of 7×10^5^ cells per plate and allowed to attach for 24 h before drug treatment. Following drug treatment (16 h), cells were lysed in Triton X-100 lysis buffer (25 mM Tris-HCl pH 7.5, 100 mM NaCl, 2.5 mM EGTA, 2.5 mM EDTA, 20 mM NaF, 1 mM Na_3_VO_4_, 10 mM sodium pyrophosphate, 20 mM sodium β-glycerophosphate, 0.5% Triton X-100) containing freshly added protease inhibitor cocktail (Roche Diagnostics, Mannheim, Germany) and the lysates were centrifuged at 13000 rpm for 10 min at 4°C. Supernatant was separated and the pellet was solubilized in SDS lysis buffer (65 mM Tris-HCl pH 7.5, 150 mM NaCl, 2% SDS) before sonication. The supernatant was defined as the soluble tubulin fraction and the solubilized pellet was defined as the insoluble tubulin fraction. Protein contents of the soluble tubulin fractions were determined using a modified Bradford assay (Bio-Rad laboratories) while that of the insoluble tubulin fractions were analyzed using BCA protein assay kit (Pierce) as described in the manufacturer’s manual.

### Cell Lysate Preparation for Total Tubulin (Containing Soluble and Insoluble Fractions)

HCT 116 (7×10^5^) cells were grown on 60 mm plates and incubated for 24 h before drug treatment. Cycloheximide (CHX) was added as a protein synthesis inhibitor along with the cinnamaldehydes and the treated cells were lysed in SDS lysis buffer. Protein contents of the lysates were analyzed using BCA protein assay kit.

### BIAM Labeling Assay

Tubulin was labeled with BIAM to investigate direct interaction of tubulin with cinnamaldehydes. Briefly, purified porcine brain tubulin (4 µM) was incubated with either DMSO, CAC (100 µM), or cinnamaldehydes (50 µM and 100 µM) at 37°C for 30 min in 0.1 M PIPES-KOH and 0.5 mM MgCl_2_ pH 7.5. After incubation, 5 µl aliquots were withdrawn from the samples and incubated with 25 µl of freshly prepared 20 µM BIAM solution in 0.1 M PIPES-KOH and 0.5 mM MgCl_2_ pH 6.5 at 37°C for 15 min. The reaction was quenched using freshly prepared iodoacetamide solution (final concentration 50 mM). Protein samples were subjected to SDS-PAGE and Western blotting with HRP–conjugated streptavidin using enhanced chemiluminescence system. Equal loading of protein was confirmed by immunoblotting using anti-β-tubulin antibody.

### Densitometric and Statistical Analysis

Image J software (NIH) was used for quantification of intensities of western blot bands. Numerical data were presented as mean±SD. Statistical significance between control and treatment groups was determined using Student’s *t*-test and values of *p*<0.05 were considered as statistically significant.

## Results

### Inhibitory Effects of Cinnamaldehydes on Proliferation and Anchorage-independent Growth of HCT 116 and MCF-7 Cells

FBCA is a “hybrid analog” of BCA and FHCA that contains both the 2-benzoyloxy and 5-fluoro substituent (Fig. 1). Evaluation of its antiproliferative activity against human colon-derived HCT 116 and mammary-derived MCF-7 carcinoma cells using MTT cell viability assay revealed that FBCA expectedly displayed cytotoxic potency around 7-fold greater than naturally occurring CA (Table 1). In addition, FBCA exhibited anti-tumor potency stronger than BCA and comparable to FHCA (Table 1, GI_50_ and LC_50_ values for CA, BCA and FHCA were previously reported in [20]; presented here for comparison with antiproliferative activity of FBCA as determined in this study). BCA, FHCA and FBCA also inhibited proliferation of MRC-5 lung fibroblasts with GI_50_ and LC_50_ values 2 to 10 fold greater than that obtained for the more susceptible HCT 116 cell line. The growth-inhibitory properties of selected cinnamaldehydes were investigated in anchorage-independent cultures. Reduction in the number of cell colonies of HCT 116 or MCF-7 cells upon treatment with cinnamaldehydes was evaluated. A significant dose-dependent decrease in the number of colonies was observed in cinnamaldehyde-treated wells as compared to DMSO control. Notably, BCA, FHCA and FBCA required a much lower concentration than CA to elicit a similar effect. The negative control CAC that is devoid of the α,β-unsaturated carbonyl group did not show any reduction in the number of colonies as compared to DMSO control (Fig. 2).

### Induction of G_2_/M phase Arrest by Cinnamaldehydes

Cell cycle analyses were performed using flow cytometry to examine if the cell cycle distribution profiles of HCT 116 cells were affected by cinnamaldehydes as a manifestation of their antiproliferative action. In general, DNA histograms of the cinnamaldehyde-treated cells showed a profound increase in the proportion of cells in G_2_/M phase at the 12 and 24 h timepoints (Fig. 3a). For lethal concentrations (60 and 80 µM CA; 20 and 30 µM BCA; 10, 20 and 30 µM FHCA and FBCA), as time progressed from 24 h to 36 h of drug exposure, the accumulated G_2_/M population decreased, accompanied with an appearance of cells in the sub G_1_ phase (Fig. 3a, 3b). Interestingly, although non-lethal concentrations of the compounds (20 and 40 µM CA; 5 and 10 µM BCA; 5 µM FHCA and FBCA) also brought about arrest of cells at G_2_/M phase at an earlier time of exposure (12 h), the DNA histograms at a later time (36 h) were restored to a normal cell cycle distribution. The inactive analog CAC produced no observable effects on the cell cycle at all timepoints (Fig. 3a). To determine whether cells were arrested in G_2_ and blocked from entering mitosis or cells were arrested in M phase and prevented from completing mitosis, lysates of HCT 116 cells treated with cinnamaldehydes or paclitaxel at proapoptotic doses for 12 h were analyzed by western blotting for levels of mitosis marker phospho-histone H3. As shown in Fig. 3c, while cells treated with paclitaxel (a prototypical microtubule targeting agent that arrests cells in M phase) caused a sharp increase in phospho-histone H3, those treated with cinnamaldehydes expressed comparable levels of phospho-histone H3 as DMSO control. Similar results were observed using immunofluorescence microscopy, where percentage of paclitaxel-treated cells stained by anti-phospho-histone H3 antibody was significantly increased (22.0% for paclitaxel *versus* 2.3% for DMSO control). In contrast, treatment with cinnamaldehydes did not increase the percentage of phospho-histone H3-positive cells (Fig. 3d). These results therefore suggested that cinnamaldehydes caused arrest of cells in G_2_ phase and cells were prevented from entering mitosis.

### Dysregulation of Cell Cycle Regulatory Proteins Mediated by Proapoptotic Doses of Cinnamaldehydes

As cdk1 has been described as one of the master regulators of mitosis [22], it was deduced that cinnamaldehydes might affect G_2_/M phase progression through dysregulation of cdk1. For investigation, a lethal concentration of cinnamaldehydes (80 µM for CA, 30 µM for BCA, FHCA and FBCA) that led G_2_ arrested HCT 116 cells to cell death was selected to examine the possible effects on cdk1 in relation to induction of tumor cell death. It was found that levels of phospho-cdk1 as well as total cdk1 declined in a time-dependent manner; the decrease in total cdk1 was more marked than that of the phosphorylated protein (Fig. 4). Densitometric analysis of western blot bands from independent experiments was performed; notably, for FHCA and FBCA, this time-dependent decline was correlated to the onset of apoptosis around 16 h, which was indicated by the appearance of cleaved forms of caspase 3 (17 and 19 kDa) and PARP (89 kDa) (lower panels of western blot analyses for each compound). For BCA and naturally occurring CA, similar correlation of decline in phospho-cdk1 and total cdk1 with emergence of cleaved PARP and caspase 3 was observed at 16 to 24 h of drug treatment. Following onset of apoptosis, cellular events such as proteolysis would have taken place as the cell progressed into late apoptosis, such that cleaved forms of caspase 3 and PARP were further cleaved to smaller peptide fragments. This might explain why at a late timepoint of 36 h, a decrease in the levels of cleaved PARP and caspase 3 was observed for BCA, FHCA and FBCA (Fig. 4b–d). On this note, we do not rule out the possibility that decline in levels of proteins at 24 to 36 h upon drug treatment could be a cellular consequence of late apoptosis. In addition, as cyclin B1 is also involved in regulating G_2_ to M transition [23], effects of cinnamaldehydes on total and phosphorylated forms of this protein were also analyzed. The levels of phospho-cyclin B1 and total cyclin B1 were found to increase upon 3 h of cinnamaldehyde treatment, remained elevated till 24 h, and declined by 36 h (Fig. 4). Consistent with results presented in Fig. 3c and 3d, levels of phospho-histone H3 remained constant to levels at 0 h, until a mild decline was observed at 24 and 36 h.

The dose-dependent effect of cinnamaldehydes on proteins regulating the G_2_/M phase was also evaluated at two timepoints: at 12 h where onset of apoptosis was triggered, and at 24 h where late apoptosis had likely set in. As shown in Fig. 5, lethal doses of cinnamaldehydes decreased the levels of total cdk1 and phospho-cdk1 (Tyr15), with the former declining more markedly as compared to that of the latter. At these doses, levels of total cdc25C as well as phospho-cdc25C (Ser216) also decreased. Conversely, cyclin B1 levels were upregulated with a more apparent increase at 24 h. Interestingly, at 12 h, the levels of cyclin B1 in both untreated control and cinnamaldehyde-treated cells were generally higher than those at 24 h. One proposed plausible explanation could be that cells were in exponential growth phase at 12 h of drug treatment. By 24 h, cells began to exit out of exponential growth, which might contribute to lower levels of cyclin B1. Nonetheless, it remains to be further investigated why levels of cyclin B1, but not other cell cycle proteins, produced more apparent differential levels of expression between the two timepoints. Finally, levels of mad2, cdc20 and survivin, the proteins that regulate proper alignment of sister chromatids to the mitotic spindle, were found to be decreased at these concentrations (Fig. 5a–d).

To exclude the possibility that decline in cell cycle regulatory proteins was a result of early apoptosis that occurred around 12 to 16 h of cinnamaldehydes treatment, cells pretreated with 50 µM pan caspase inhibitor Z-VAD-FMK for 1 h were treated with the more cytotoxic analog FBCA for 12 h. Under the cellular circumstances where apoptosis was blocked (evident from the absence of cleaved PARP and caspase 3), decline in the levels of total cdk1, phospho-cdk1, total cdc25C and phospho-cdc25C was still apparent and concurred with levels observed in cells untreated with Z-VAD-FMK (Fig. 6). The results therefore suggested that loss of these cell cycle regulatory proteins was an effect of the drugs and not a consequence of cell death.

### Interaction of Tubulin with Cinnamaldehydes Leading to Tubulin Aggregation

On the basis that α,β-unsaturated carbonyl compounds have been demonstrated to display affinity for sulfhydryl groups in tubulin [24], interaction of Michael acceptor-bearing cinnamaldehydes with tubulin was investigated using purified tubulin. After incubation with cinnamaldehydes, tubulin samples were incubated with BIAM, which would label free thiol groups in the protein. Modification of thiol sites by cinnamaldehydes would render them unavailable for BIAM labeling, observed as a reduction in the intensities of bands probed with HRP-streptavidin. Indeed, a marked decrease was observed in the band intensities of cinnamaldehydes-treated samples as compared to DMSO control while CAC failed to reduce labeling by BIAM (Fig. 7a). In agreement with the antiproliferative activities, CA displayed a weaker interaction with the sulfhydryl groups in tubulin as compared to BCA, FHCA and FBCA. The possible effect of cinnnamaldehydes binding to cellular tubulin was next investigated. HCT 116 cells treated with lethal concentrations of cinnamaldehydes were immunolabelled for tubulin. Fluorescence microscopic images revealed conspicuous tubulin aggregation and loss of the interphase microtubular network (Fig. 7b). To further examine the tubulin aggregation phenotype, we assessed the distribution of soluble and insoluble tubulin upon drug treatment by analyzing the soluble and insoluble cell lysate fractions. The soluble fraction contained the dimeric α,β-tubulin while the insoluble fraction contained the tubulin polymers and aggregates. The results had agreed with the immunocytochemistry microscopic observations; in cells that were exposed to cinnamaldehydes for 16 h, a dose-dependent increase in the insoluble tubulin content was observed along with depletion of tubulin in the soluble fraction (left and middle panel of Fig. 8a). Densitometric analysis of western blots obtained from independent experiments revealed a statistical significant accumulation of insoluble tubulin normalized to actin for cytotoxic concentrations of cinnamaldehydes that were previously determined to progress G_2_/M arrested cells to cell death (right panel of Fig. 8a). Decline in soluble tubulin had appeared to be gradual in contrast to the marked increase in insoluble tubulin. We reasoned that at equilibrium, cellular tubulin largely existed in the soluble phase. As a result, relative to changes in insoluble tubulin, changes in soluble tubulin content would be marginally small and less significantly detected on western blots. The possibility of cinnamaldehydes affecting tubulin degradation was also examined. A time-chase experiment was performed in which cells were treated with BCA alongside protein synthesis inhibitor cycloheximide (CHX). Both CHX plus BCA-treated cells and CHX only-treated cells did not display any changes in the total tubulin content with respect to time, indicating that degradation of tubulin was not affected by cinnamaldehydes (Fig. 8b).

## Discussion

Previous studies have reported anti-tumor properties of CA and its analogs in mammalian cancer cell lines as well as in mouse xenograft models [8], [10]–[13], [16]–[19]. Although the studies report wide-ranging molecular effects of cinnamaldehydes, the precise mechanisms by which cinnamaldehydes elicit their anti-cancer properties remain obscure. It is believed that cinnamaldehydes exert their anti-tumor effects via several mechanisms of action. In a previous study conducted in our laboratory, BCA and FHCA were identified as the lead analogs possessing potent antiproliferative activities; the enhancement in biological activity was attributed to the electron withdrawing 2-benzoyloxy and 5-fluoro substituent in BCA and FHCA respectively [20]. In this study, in an attempt to derive a structural analog that would possess the desirable characteristics of BCA and FHCA, a novel analog FBCA that bears both 2-benzoyloxy and 5-fluoro substituents was synthesized and evaluated along with BCA and FHCA. In the MTT assay, FBCA expectedly demonstrated superior and comparable anti-tumor properties against HCT 116 and MCF-7 cells as compared to BCA and FHCA respectively. Similarly, in the soft agar assay, the superior antiproliferative activity of FBCA was further demonstrated as a dose-dependent reduction in the anchorage-independent growth of HCT 116 and MCF-7 cells.

The G_2_/M phase arrest brought about in HCT 116 cells upon treatment with cinnamaldehydes at sub-lethal and lethal doses culminated in a different cellular outcome. For the lethal concentrations used, as time progressed, the G_2_/M cell population declined with a corresponding increase in the sub G_1_ population, whose appearance would indicate that the G_2_/M arrested cells proceeded to cell death. The lethal doses failed to increase levels of mitosis marker phospho-histone H3, indicating that cinnamaldehydes arrested cells at G_2_ phase and not the M phase. On the other hand, the sub-lethal concentrations caused a transient arrest of cells at G_2_/M phase, which decreased at a later timepoint (36 h) with the restoration of a normal cell cycle distribution. The lack of appearance of the sub G_1_ cell population indicated that the cells survived the episode of drug insult, likely to be through activation of cellular repair mechanisms to relieve the cells out of G_2_/M phase arrest.

Examination of the effect of cinnamaldehydes on the cell cycle regulatory proteins responsible for controlling G_2_ to mitosis transition and mitotic spindle formation had found a drug-mediated dose- and time-dependent decline in the levels of phospho-cdk1 and total cdk1, as well as a dose-dependent increase in the levels of phospho-cyclin B1 and total cyclin B1. The total cdk1 levels showed a more pronounced decline than the phospho-cdk1 (Tyr15), indicating that cinnamaldehyde-treated cells might contain a net increase in the phosphorylated form of cdk1 as compared to the control cells. An accumulation of cyclin B1 had resulted, while total cdc25C and phospho-cdc25C (Ser216), which is upstream of cdk1 in the activation cascade, were dose-dependently down-regulated in response to drug treatment. As cdc25C dephosphorylates the inactive phospho-cdk1 into the active cdk1 [25], a reduction in the levels of cdc25C would likely explain the higher levels of phospho-cdk1. Western blot analyses showed that the appearance of cleaved forms of caspase 3 and PARP was concomitant with cdk1 down-regulation. As the role of cdk1 is essential throughout mitosis, we construed that blocking cdk1 activation by cinnamaldehydes had in part led to the arrest of cells at G_2_ phase. Cyclin B1 complexes with cdk1 and during late G_2_ phase, and activation of the cdk1/cyclin B1 complex through cdc25C-dependent dephosphorylation of phospho-cdk1 and phosphorylation of cyclin B1 triggers cells to enter mitosis [24]. Cinnamaldehydes had caused accumulation of cyclin B1, which was not coupled with accumulation of non-phosphorylated cdk1. Thus, it was deduced that cdk1/cyclin B1 complexes failed to be activated; cells in G_2_ phase were prevented from entering mitosis, which eventually triggered apoptotic cell death.

Formation of the microtubular mitotic spindle is crucial in mitosis for proper segregation of chromosomes [4]. Cells check for unattached chromosomes or any damages to the spindle before proceeding to anaphase and activate the spindle assembly checkpoint if any misalignment or improper attachment is detected [26]. We had examined the levels of proteins regulating spindle assembly such as mad2, cdc20 and survivin. Cdc20 is a co-factor for the ubiquitin ligase APC/C which degrades proteins like cyclin B and securin to allow the cell to proceed into anaphase. However, in case of misalignment or improper spindle formation, cdc20 is inhibited by proteins such as mad2 to allow for appropriate alignment [27]. Survivin is a member of the inhibitor of apoptosis (IAP) family, and has dual roles in mitosis and apoptosis. Expression of survivin increases during mitosis where it associates with microtubules [28]. It also acts as an inhibitor of apoptosis and its expression has been associated with aggressive tumors [29]. Down-regulation of survivin is therefore considered as a potential strategy for cancer therapy [30]. Upon treatment with cinnamaldehydes, both mad2 and cdc20 were depleted at the lethal concentrations. Survivin levels also declined at these concentrations of cinnamaldehydes, thus weakening the anti-apoptotic machinery and rendering the cells more susceptible to apoptosis. Again, it was noted that apoptosis, as detected by appearance of cleaved caspase 3 and cleaved PARP, concurred with the doses at which the cell cycle regulatory proteins were altered. This had indicated that the induction of apoptosis was associated with dysregulation of cell cycle regulatory proteins.

Microtubules are cytoskeletal components that are composed of α- and β-tubulin heterodimers, of which a number of isotypes of the human α and β protein exist. Microtubules and their tightly regulated polymerization dynamics during mitosis are targets for microtubule-targeting agents (MTAs) [31]. The MTAs such as taxanes (paclitaxel and docetaxel) and Vinca alkaloids (vincristine and vinblastine) arrest cells at M phase by direct binding to microtubules to disrupt microtubule dynamics [6], [7]. In recent years, compounds of diversified structures are discovered to target tubulin or microtubules with different mechanisms of action. For example, peroxisome proliferator-activated receptor γ (PPAR-γ) inhibitors are found to reduce tubulin levels likely through proteasomal degradation without affecting microtubule polymerization or dynamics [32], [33]. On the other hand, isothiocyanates (ITCs) are found to cause formation of tubulin aggregates through direct binding to cysteine residues in tubulin, followed by tubulin degradation in a proteasomal-dependent manner [34]. We had found that cinnamaldehydes did not alter the tubulin polymerization kinetics *in vitro* (data not shown), suggesting the possible involvement of other cellular machinery. Consistent with work by Ishiguro et al. that reported the reaction of α,β-unsaturated carbonyl compounds with sulfhydryls of tubulin [22], we found *in vitro* evidence that cinnamaldehydes containing α,β-unsaturated carbonyl groups interacted with tubulin protein. The interaction had likely led to the tubulin aggregation phenotype as observed using immunofluorescence microscopy. These tubulin aggregates contributed to dose-dependent accumulation of insoluble tubulin. However, tubulin degradation was not affected, indicating that cinnamaldehydes appeared to be targeting tubulin with a mechanism of action distinct from the current MTAs. The imbalanced levels of soluble and insoluble tubulin might have halted proper microtubule polymerization dynamics. This could at least in part contribute to blockade of cells to enter M phase, thus leading to a G_2_ arrest.

To conclude, in this study, CA and its analogs (BCA, FHCA ad FBCA) with superior antiproliferative activities were found to cause reduction in levels of cell cycle proteins such as cdk1, cdc25C, mad2, cdc20 and survivin, elevation in levels of cyclin B1, and aggregation of tubulin. Decline in cdk1 and cdc25C levels, as well as tubulin aggregation contributed at least in part to arresting cinnamaldehyde-treated cells in G_2_ phase, which led to apoptotic cell death (summarized in Fig. 9). As cells were prevented from entering mitosis, dysregulation of levels of spindle assembly regulatory proteins mad2, cdc20 and survivin, which would result in defective mitotic spindle formation, could not have taken place. Thus, it is unlikely that depletion of these proteins regulating spindle assembly accounts for cinnamaldehyde-induced cell death. Generally, it is believed that multiple molecular targets underscore the antiproliferative activities of cinnamaldehydes. While ongoing work continues to map out the precise network of mechanisms of anti-tumor action of this class of compounds, this study had produced experimental findings supporting the notion that cinnamaldehydes induced G_2_ arrest and cell death that was at least in part associated with tubulin targeting and reduction in levels of cell cycle regulatory proteins like cdk1 and cdc25C. Given that cell cycle proteins and tubulin are essential for all cells, particularly proliferative cells, cinnamaldehydes may exert toxicities on normal cells. Indeed, we had observed antiproliferative effects of these analogs on normal lung MRC-5 fibroblasts with potencies 2 to 10 times lower than that on the susceptible HCT 116 cell line. Therefore, development of analogs that show preferential action on cell cycle reglulatory proteins and tubulin in tumor cells and/or screening of selective sensitivity of these proteins in tumors of individual patients are warranted for the potential clinical application of this chemical class of compounds.
